# Status Epilepticus as a Novel Late Complication in CARASIL: A Case Report Expanding the Clinical Spectrum

**DOI:** 10.1002/ccr3.72510

**Published:** 2026-04-19

**Authors:** Mohammad Amin Najafi, Seyyed Ali Alaei, Alireza Anjam Majoumerd, Seyyed AmirHossein Mirghanizadeh Bafghi, Mahdi Vajdi, Mohammad Reza Najafi

**Affiliations:** ^1^ Department of Neurology Alzahra Hospital, Isfahan University of Medical Sciences Isfahan Iran; ^2^ School of Medicine Isfahan University of Medical Sciences Isfahan Iran; ^3^ Department of Clinical Nutrition, School of Nutrition and Food Science Isfahan University of Medical Sciences Isfahan Iran

**Keywords:** CARASIL, HTRA1 variant, leukoencephalopathy, small vessel disease, status epilepticus

## Abstract

CARASIL may present without systemic features, and seizures including status epilepticus can occur as late‐stage complications. Long‐term neurological monitoring is essential even in patients lacking extra‐neurological manifestations.

## 1 Introduction

Cerebral autosomal recessive arteriopathy with subcortical infarcts and leukoencephalopathy (CARASIL) is an exceptionally rare, non‐hypertensive, autosomal recessive cerebral small vessel disease caused by pathogenic variants in the high‐temperature requirement protease A1 (HTRA1) gene. It is typically characterized by recurrent subcortical infarcts, progressive cognitive decline, alopecia, and spondylosis [1]. Seizures, although uncommon, have been reported as a possible clinical manifestation in patients with CARASIL [2]. Here, we describe a genetically confirmed case of CARASIL due to a pathogenic variant in HTRA1, notable for the absence of extra‐neurological features such as alopecia or spondylosis, who presented with status epilepticus 17 years after the onset of initial CARASIL symptoms.

## 2 Case History/Examination

**FIGURE 1:**
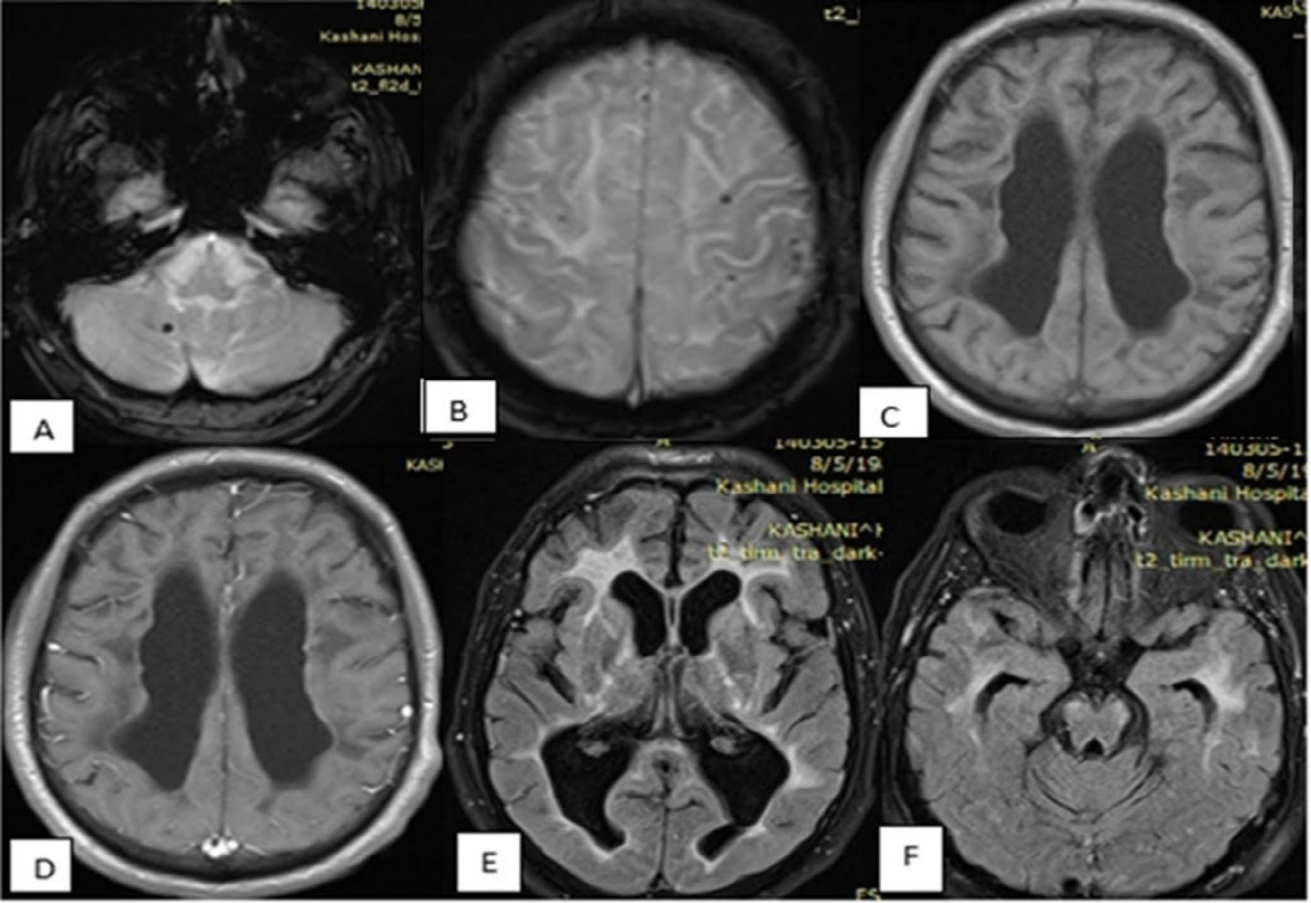
Brain MRI of the patient at admission. (A, B) Gradient echo sequences showing focal low signal intensities suggestive of cerebral microhemorrhages. (C) T1‐weighted image demonstrating hypointensities in the periventricular and subcortical regions. (D) Post‐contrast T1‐weighted image showing no pathological enhancement. (E, F) FLAIR sequences revealing confluent hyperintensities in the periventricular and subcortical white matter, anterior temporal lobes, and external capsule, consistent with diffuse leukoencephalopathy and associated brain atrophy.

**FIGURE 2:**
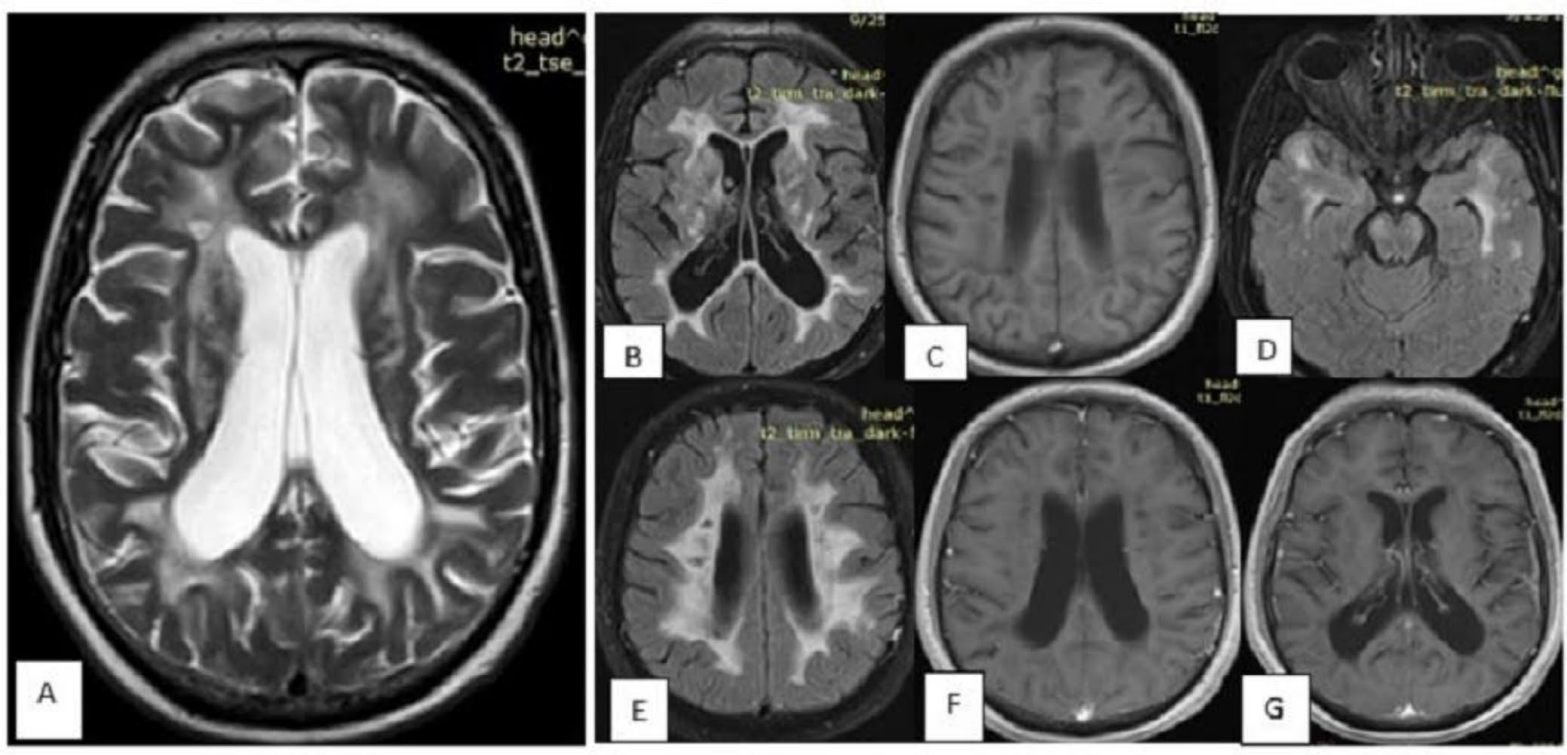
Initial brain MRI of the patient at age 23. (A) T2‐weighted image showing confluent hyperintensities in the periventricular and subcortical white matter. (B–D) FLAIR sequences demonstrating confluent hyperintensities in the periventricular, subcortical, anterior temporal, and external capsule regions, consistent with early diffuse leukoencephalopathy. (E) T1‐weighted image without contrast showing corresponding hypointensities. (F) Post‐contrast T1‐weighted image showing no abnormal enhancement.

**FIGURE 3:**
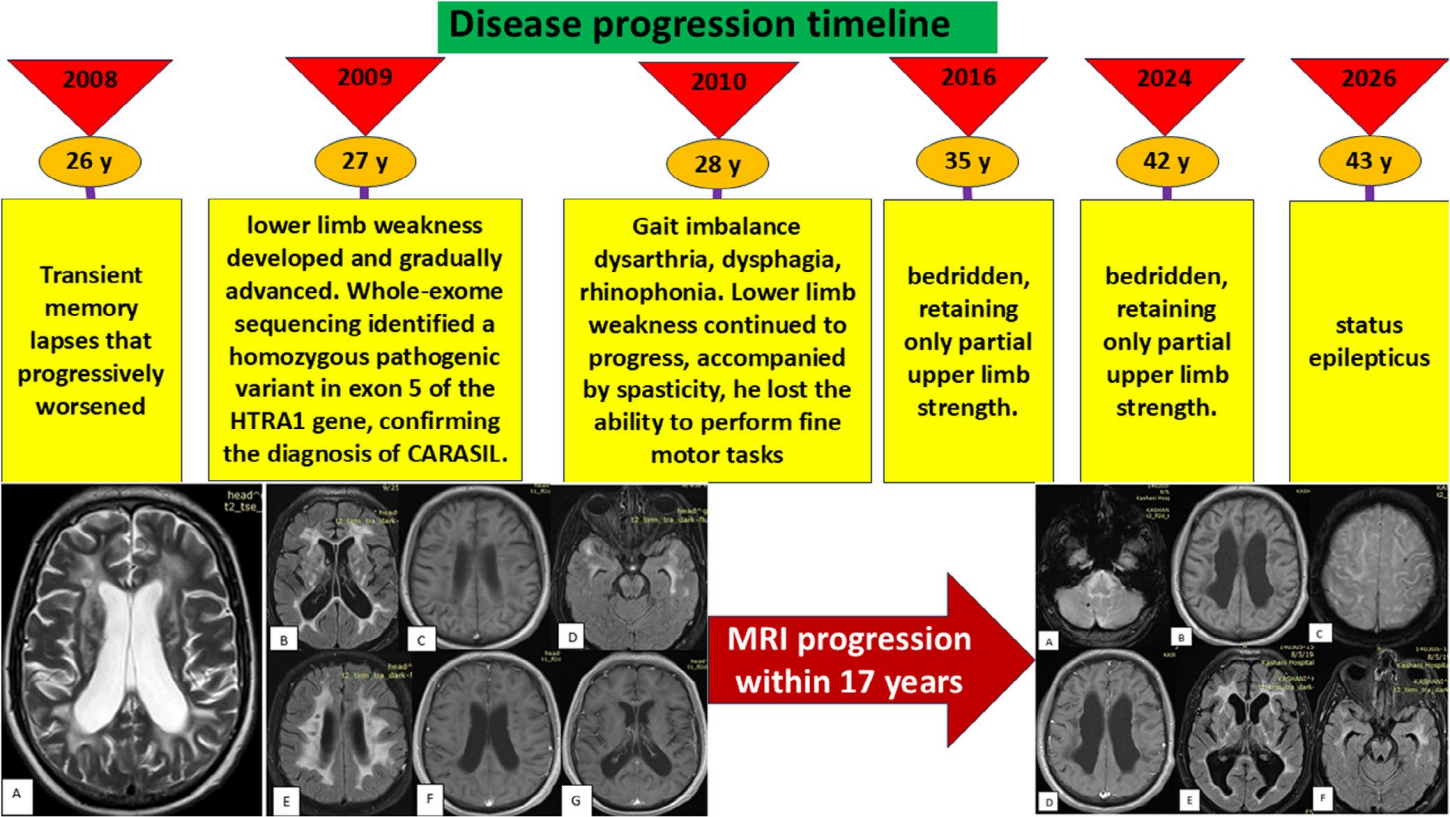
Schematic timeline summarizing the disease progression.

A 43‐year‐old man with a genetically confirmed diagnosis of CARASIL was admitted to our emergency department with status epilepticus. He was intubated and managed with a continuous midazolam infusion in combination with high‐dose antiepileptic therapy, including sodium valproate, levetiracetam, and phenytoin. Seizure activity was subsequently controlled, and the patient gradually regained consciousness. The midazolam infusion was discontinued, and antiepileptic medications were tapered and transitioned to oral administration. Brain MRI revealed confluent T2/FLAIR hyperintensities in the periventricular, subcortical, anterior temporal, and external capsule regions, consistent with diffuse leukoencephalopathy, as well as low signal intensity on gradient‐echo sequences, suggestive of microhemorrhage (Figure 1). The patient's initial symptoms began at age 26 with transient memory lapses that progressively worsened. Within 1 year, lower limb weakness developed and gradually advanced. At age 27, his first brain MRI was performed (Figure 2). Seven months later, gait imbalance emerged, followed by bulbar symptoms at age 28, including dysarthria, dysphagia, and rhinophonia. Lower limb weakness continued to progress, accompanied by bradykinesia and spasticity, and he lost the ability to perform fine motor tasks such as buttoning clothes. By age 35, the patient had become bedridden, retaining only partial upper limb strength. Approximately 9 months prior to the current admission, he experienced two episodes of generalized tonic–clonic seizures characterized by jaw clenching and frothing at the mouth. The first seizure lasted 15 min, followed by a second episode 5 min later that lasted 2 min. Since then, he had been maintained on sodium valproate 750 mg daily in two divided doses (Figure 3, summarized the timeline of disease progression).

## 3 Differential Diagnosis

Given the progressive neurological deterioration and characteristic neuroimaging findings—including confluent white matter hyperintensities, lacunar infarcts, cerebral microhemorrhages, and involvement of the anterior temporal lobes and external capsule—a structured differential diagnosis encompassing both hereditary and acquired cerebral small vessel diseases was pursued. Inflammatory demyelinating disorders, vasculitis, mitochondrial encephalopathies, thrombophilia, and adult‐onset leukodystrophies were considered. Cerebrospinal fluid analysis revealed normal cell counts, protein, and glucose levels, with oligoclonal bands and lactate within normal limits. Serum inflammatory and autoimmune panels—including antinuclear antibodies (ANA), anti‐neutrophil cytoplasmic antibodies (ANCA), anti–double‐stranded DNA antibodies, antiphospholipid antibodies, and erythrocyte sedimentation rate (ESR)—were negative. Screening for neuromyelitis optica spectrum disorder (anti–aquaporin‐4 [AQP4] antibodies) and myelin oligodendrocyte glycoprotein (MOG) antibody disease was also negative. Both inherited and acquired thrombophilic conditions were systematically evaluated and excluded through a comprehensive thrombophilia panel, including Factor V Leiden (polymerase chain reaction [PCR]), prothrombin gene mutation, homocysteine, protein C, protein S, and antithrombin III, which showed no abnormalities. Metabolic and nutritional causes were excluded by normal serum levels of vitamin B12, zinc, copper, and arsenic. Infectious etiologies, including HIV and neurosyphilis, were ruled out by negative serology. Given the early adult onset, progressive course, and imaging features suggestive of hereditary small vessel disease, genetic testing was pursued. Whole‐exome sequencing was performed on a targeted next‐generation sequencing panel, followed by confirmatory Sanger sequencing to validate candidate variants. This revealed a homozygous likely pathogenic variant in exon 5 of the HTRA1 gene, with full pathogenicity assessment conducted according to the 2015 American College of Medical Genetics and Genomics (ACMG) guidelines. The identified variant is [HTRA1 (NM_002775.5): c.982G>A; p.(Ser328Asn)], confirming the diagnosis of cerebral autosomal recessive arteriopathy with subcortical infarcts and leukoencephalopathy (CARASIL).

The pathogenicity of the c.982G>A (p.Ser328Asn) variant was evaluated based on ACMG/AMP criteria [3]. Based on the cumulative evidence, the variant is classified as Likely Pathogenic according to ACMG guidelines. No pathogenic variants were identified in NOTCH3, effectively excluding CADASIL. The genetic findings, in conjunction with the clinical phenotype and neuroimaging characteristics, established the final diagnosis.

## 4 Conclusion and Results (Outcome and Follow‐Up)

This case illustrates a rare phenotype of genetically confirmed CARASIL, distinguished by the absence of extra‐neurological features and the late emergence of status epilepticus, occurring 17 years after the initial symptom onset. The identified HTRA1 c.982G>A variant was interpreted as likely pathogenic based on genetic testing results and its consistency with the patient's clinical phenotype.

These observations broaden the recognized clinical spectrum of CARASIL and underscore the need to consider seizures, including status epilepticus, as potential complications in advanced stages of the disease. Continuous long‐term surveillance for neurological decline, even in patients lacking systemic manifestations, remains crucial in the management of CARASIL.

## 5 Discussion

CARASIL is a genetically related non‐hypertensive cerebral small vessel arteriopathy that mainly manifests with progressive cognitive impairment and recurrent stroke with subcortical infarcts in early adulthood [1, 4, 5, 6, 7]. Non‐neurological manifestations of CARASIL include alopecia and spondylosis [3, 6]. Arteriosclerosis, loss of vascular smooth muscle, and hyaline degeneration of the tunica media are among the pathologic hallmarks of CARASIL [4]. Pathogenic variants in *HTRA1*, as observed in our case, lead to reduced HTRA1 protease activity, resulting in dysregulated transforming growth factor–β signaling, excessive extracellular matrix deposition, and progressive vascular fibrosis [5, 8].

The pathogenicity of the identified HTRA1 variant c.982G>A; p.(Ser328Asn) was evaluated in accordance with the ACMG guidelines [3]. This missense variant affects a conserved amino acid residue within the HTRA1 protease domain, supporting moderate evidence for pathogenicity. In silico predictive tools suggest a deleterious impact on protein function, providing supporting evidence. In addition, the variant is consistent with the patient's clinical phenotype and radiological findings characteristic of CARASIL. Although functional studies and segregation analysis are not available, the overall body of evidence—including genotype–phenotype concordance—supports the classification of this variant as likely pathogenic, in agreement with the genetic testing report.

While some limitations remain, including the lack of functional validation and limited published data on this specific variant, the convergence of clinical, radiological, and genetic findings strengthens its presumed disease‐causing role. Therefore, this variant is best interpreted as likely pathogenic rather than a Variant of Uncertain Significance (VUS) in this clinical context.

Prior studies indicate that the extra‐neurological system manifestations, such as alopecia and spondylosis, can distinguish CARASIL from other CVSDs [3, 6]. However, recent reports have documented cases of CARASIL without these classic features, particularly when the disease is identified through genetic testing rather than clinical presentation alone [9]. Most reported cases exhibit rapid neurological deterioration, with severe disability or death occurring within the first decade after symptom onset [7]. Compared with previously reported CARASIL cases, our patient demonstrates a relatively prolonged clinical course, with survival extending 17 years after symptom onset, which is longer than what is typically described in the literature.

Our patient's first symptoms occurred 17 years before the current admission, which showed relatively higher survival and had a course of progression without extra‐neurological symptoms and signs. Our case also manifests with progressive cognitive impairments, but recent GTC seizures and recent status epilepticus presentation after 17 years of first symptoms in our case were a more unique neurological characterization for CARASIL, alongside the absence of manifestations like alopecia and spondylosis. There is limited data on seizure pathophysiology and prognosis in CARASIL, but few reports have mentioned it. Tan et al. [10] described a CARASIL case with GTC seizures, where the patient developed alopecia in his 20s, experienced cognitive decline in his 30s, and had GTC seizures in his 40s before becoming bedridden and passing away at the age of 46. Kumar et al. [11] had described a 24‐year‐old male with CARASIL who presented with personality and behavioral changes lasting 3 years, lower limb spasticity for 2 years, memory impairment, executive function deficits, and mutism for the past 6 months. This patient ultimately died from status epilepticus. Another study by Devaraddi et al. [12] reported a 27‐year‐old male with CARASIL who presented with a stroke, neuropsychiatric symptoms, and episodes of seizures starting at the age of 25. When these cases are compared with our patient, seizure occurrence appears to emerge after long‐standing disease progression rather than at early stages.

We hypothesize that two potential pathophysiological mechanisms may explain the etiology of seizure activity. First, while CARASIL is predominantly a white matter disorder, progressive cortical atrophy and neuronal loss, particularly in the frontal and temporal regions, may develop over time, potentially contributing to epileptogenesis. Second, microhemorrhages and hemosiderin deposition, evidenced by low signal intensity on GRE/T2* sequences in our patient, suggest cerebral microbleeds—a hallmark of small vessel disease. Cortically situated microhemorrhages could serve as epileptogenic foci, further increasing seizure susceptibility. Taken together, these findings suggest that status epilepticus may represent a late‐stage neurological complication of advanced CARASIL rather than an early disease manifestation.

## Author Contributions

**Mohammad Amin Najafi:** conceptualization, methodology, visualization, writing – original draft, writing – review and editing. **Seyyed Ali Alaei:** data curation, investigation, methodology, writing – original draft. **Alireza Anjam Majoumerd:** data curation, investigation, writing – original draft, writing – review and editing. **Seyyed AmirHossein Mirghanizadeh Bafghi:** data curation, investigation, methodology, validation, writing – original draft, writing – review and editing. **Mahdi Vajdi:** data curation, writing – original draft, writing – review and editing. **Mohammad Reza Najafi:** conceptualization, methodology, project administration, supervision, validation, visualization.

## Funding

The authors have nothing to report.

## Consent

Written informed consent was obtained from the patient.

## Conflicts of Interest

The authors declare no conflicts of interest.

## Data Availability

Data sharing is not applicable to this article, as no datasets were generated or analyzed in the current study.
